# Assessing the experience and attitude of emergency medical services staff toward linguistic diversity challenges in a Middle Eastern pre-hospital emergency care environment using machine learning analysis methods

**DOI:** 10.5339/qmj.2025.19

**Published:** 2025-03-03

**Authors:** Hassan Farhat, Guillaume Alinier, Ian Howland, Houcine Kanoun, Mohamed Chaker Khenssi, Loua Al Shaikh, James Laughton

**Affiliations:** ^1^Ambulance Service, Hamad Medical Corporation, Doha, Qatar; ^2^Faculty of Medicine “Ibn El Jazzar”, University of Sousse, Sousse, Tunisia; ^3^Faculty of Sciences, University of Sfax, Sfax, Tunisia; ^4^School of Health and Social Work, University of Hertfordshire, Hatfield, UK; ^5^Weill Cornell Medicine – Qatar, Doha, Qatar; ^6^Faculty of Health and Life Sciences, Northumbria University, Newcastle upon Tyne, UK *Correspondence: Hassan Farhat. Email: hassen.farhat@gmail.com

**Keywords:** Language barriers, linguistic challenges, cultural diversity, pre-hospital care, Middle Eastern EMS

## Abstract

**Background:**

Language barriers significantly impact healthcare delivery, particularly in emergency medical services (EMS) operating in linguistically diverse environments. The demographic composition of Qatar, with its predominantly expatriate population, presents unique challenges for effective communication in pre-hospital care settings. The aim of this was to assess the opinions of personnel from the Hamad Medical Corporation Ambulance Service (HMCAS) regarding the impact of language barriers on pre-hospital emergency care.

**Methods:**

A cross-sectional study was conducted using an anonymous survey with a five-point Likert scale among 312 frontline personnel of HMCAS. Fisher's exact and Kruskal–Wallis tests were used to compare ordinal outcomes across groups. Machine learning algorithms, including ordinal logistic regression, support vector machines (SVM), and naive Bayes, were used to develop predictive models for HMCAS staff opinions on their language learning needs.

**Results:**

Both bivariate and multivariate analyses revealed significant differences in the frequency of experiencing communication challenges. The most influential factors identified were strong opinions on language barriers and the willingness of staff to enhance their language skills. Variables related to using family members as interpreters showed relatively low importance. The SVM model demonstrated the best predictive capability concerning staff perceptions about language learning needs, with an accuracy of 0.50 and an average area under the curve score of 0.74.

**Conclusion:**

Language barriers significantly impact pre-hospital emergency care in Qatar. The findings highlight the need for targeted interventions, such as language training programs and mobile translation apps. These strategies could enhance communication in multicultural EMS settings, improving patient care and reducing miscommunication risks. Future research should evaluate the long-term impact of these interventions on patient outcomes.

## Introduction

Effective communication is crucial to ensure the delivery of high-quality healthcare services. In the context of patient safety culture, communication plays a vital role in enabling healthcare providers to accurately assess patient needs, convey important information, and make informed decisions. In pre-hospital settings, where emergency medical services (EMS) operate under time-sensitive and often chaotic conditions, clear and effective communication helps significantly reduce the risk of errors and enhance patient outcomes.^1,2^

Middle Eastern countries face unique challenges regarding effective communication in healthcare due to their highly diverse populations. For example, in Qatar, recent statistics indicate that expatriates constitute approximately 89.13% of the population.^3^ In 2023, a recent study indicated that 75.61% of individuals in Qatar accessed pre-hospital emergency healthcare services.^4^ The expatriate population in Qatar is notably diverse, with significant communities from South Asia and North Africa, among others.^4,5^ This demographic diversity presents challenges for EMS providers, who often encounter patients speaking different languages, thereby hindering the timely and effective care delivery.

The existing body of literature has extensively documented the impact of language barriers on healthcare delivery in various settings. Studies have shown that language discordance can lead to increased communication errors, unnecessary procedures, and higher healthcare costs.^6,7^ However, there is a scarcity of targeted research aimed at addressing these challenges for EMS in multilingual environments.

The Hamad Medical Corporation Ambulance Service (HMCAS) is a modern and leading public EMS provider in Qatar, operating in a multilingual environment.^8^ In 2024, the International Academies of Emergency Dispatch distinguished HMCAS as an “Accredited Center of Excellence (ACE)” for the fourth consecutive year.^9^ To effectively address the linguistic diversity present in Qatar, HMCAS has employed a diverse workforce proficient in multiple languages.^10,11^

The aim of this study was to assess the opinions of HCMAS frontline personnel about the impact of language barriers on pre-hospital emergency care in Qatar, using advanced statistical and computational techniques to identify potential strategies for improvement.

## Methods

### Study design

This cross-sectional study used an anonymous online survey with a five-point Likert scale, which was developed by researchers and administered between March and June 2023. Participants were required to sign an online consent form before participation. The survey comprised 13 items. It included questions on demographic information, professional experience, frequency of language barrier encounters, perceived sufficiency of patient information obtained during language barrier situations, comfort level with using bystanders as interpreters, perceived need for additional language learning, willingness to learn new languages, ability to attend language courses, and satisfaction with communication during a major sporting event. Responses were collected using a five-point Likert scale, ranging from “strongly disagree” to “strongly agree”, to assess participants’ attitudes and experiences regarding language barriers in their professional practice in HMCAS. The structure of this article followed the STROBE (Strengthening the Reporting of Observational Studies in Epidemiology) guidelines for cross-sectional studies.^12^

### Participants and sampling

This study targeted HMCAS personnel engaged in patient-facing clinical roles, specifically ambulance paramedics (APs) and critical care paramedics (CCPs). APs operate at an advanced life support level, whereas CCPs have a broader clinical scope of practice, requiring more advanced skills and responsibilities.^8,13^ Licensed HMCAS personnel, such as emergency medical dispatchers, who do not engage in direct patient interaction, were excluded from the study. This exclusion was based on the distinct factors and roles that affect their work environment, which relies solely on verbal communication, leading to a potential bias.

According to the human resources registry of HMCAS, a total of 1,115 personnel were identified as being in patient-facing roles during this study. Solvin's formula was used to determine the minimum sample size necessary for the research,^14^ resulting in a requirement of at least 295 staff members.

### Validity and reliability

A group of nine experts in paramedicine, emergency medicine, and research were invited to validate the tools. A fillable PDF form was created and sent to these experts via e-mail, enabling them to review and validate the items presented. They were asked to rate each item on a scale from one to five, with “1 = Strongly agree” and “5 = Strongly disagree”. The criteria for evaluation included pertinence, clarity of language, relevance to the objective, and the effectiveness of each item as a good indicator. Additionally, the experts were asked to provide suggestions for improvement beneath each question. Using their ratings, Kendall's W and Cohen's kappa coefficients were used to assess the content validity of the survey. Kendall's W measures the degree of agreement among multiple raters and evaluates the consistency in their ratings.^15^ In contrast, Cohen's kappa quantifies the degree of agreement between raters, adjusting for the possibility of chance agreement, thus providing a more accurate measure of inter-rater reliability in terms of content validity.^16^ Reliability was assessed using Cronbach's alpha coefficient.^14^

### Data analysis

Data analysis was conducted using the R programming language via R Studio^®^.

Data cleaning was performed to ensure consistency and ease of analysis. Subsequently, a series of analytical techniques were used, including descriptive statistics, bivariate analysis, multivariate analysis, and statistical modeling using machine learning (ML).

Firstly, mean, median, standard deviation (SD), and interquartile range (IQR) were calculated for continuous variables such as age and years of experience. Counts and percentages were calculated for categorical variables. Secondly, due to the small sample sizes and low expected frequencies observed in contingency tables, Fisher's exact test was used to examine the association between categorical variables. Thirdly, the Kruskal–Wallis test was used to compare the ordinal outcomes (Likert scale options) across multiple groups. When significant differences were found, post hoc Dunn's tests with Bonferroni correction were performed to identify group differences. Fourthly, ML techniques were used to develop predictive models for the opinions of HMCAS staff on the necessity of acquiring additional language skills. This approach facilitated an in-depth examination of the relationships between healthcare professionals’ characteristics, experiences, and attitudes towards language barriers in the HMCAS framework. Three distinct algorithms were used: ordinal logistic regression (OLR), support vector machines (SVM), and naive Bayes (NB), chosen for their ability to handle ordinal data and various classification approaches.^17^ Furthermore, the OLR is specifically designed for ordinal data analysis.^18^ Additionally, the SVM algorithm can capture complex relationships between survey items while maintaining good performance even with small sample sizes.^17^ NB was included for its simplicity, speed, and degree of effectiveness.^17^

The dataset was subsequently divided into training (70%) and testing (30%) sets to evaluate model performance. Various metrics were used to assess the performance of the models, including accuracy, kappa statistic, sensitivity, specificity, Matthews correlation coefficient, confusion matrix (CM) plots, receiver operating characteristic (ROC) curves, and the area under the curve (AUC).^19^

## Results

### Descriptive statistics

A total of 312 paramedics, mostly APs, participated in this survey, with a mean age of 36.6 years (SD 5.4). The average duration of their working years in the Middle East and North Africa (MENA) region was 8.9 years (SD 5.6), while they had spent an average of 7.2 years (SD 4.7) in a patient-facing role in Qatar. The majority of participants were of Filipino nationality (*n* = 100), followed by Tunisian (*n* = 87), Indian (*n* = 73), and Jordanian (*n* = 44), among other nationalities (Table 1 and Appendix).

Table 2 presents the results of content validity and reliability assessments. Kendall's W and Cohen's kappa coefficients were respectively equal to 0.73 and 0.69, indicating good content validity. The Cronbach's alpha coefficient was equal to 0.56, with an acceptable level of inter-item correlation, given the low number of items, as confirmed by previous research.^20^

Due to the significant differences in sample sizes between APs (n = 309) and CCPs (Table 1), Fisher's exact test was used to handle such differences. The results (Table 3) revealed a significant association between the role within HMCAS and the frequency of experiencing communication difficulties due to language barriers. Furthermore, the Kruskal–Wallis test (Table 3) showed a significant difference in communication challenges between the groups. Post hoc Dunn's tests with Bonferroni correction revealed that CCPs often experienced difficulties in communicating with patients.

The performance metrics of the ML algorithms are presented in Table 4 . Among the evaluated models, the SVM exhibited best performance across multiple evaluation metrics, achieving an accuracy of 0.50, a kappa value of 0.23, and a specificity of 0.84, which refers to the correct identification of negative instances. The SVM model also performed similarly to the OLR model, with both models displaying misclassifications in the “Maybe” and “Strongly agree” prediction categories.

**Figure 1.**
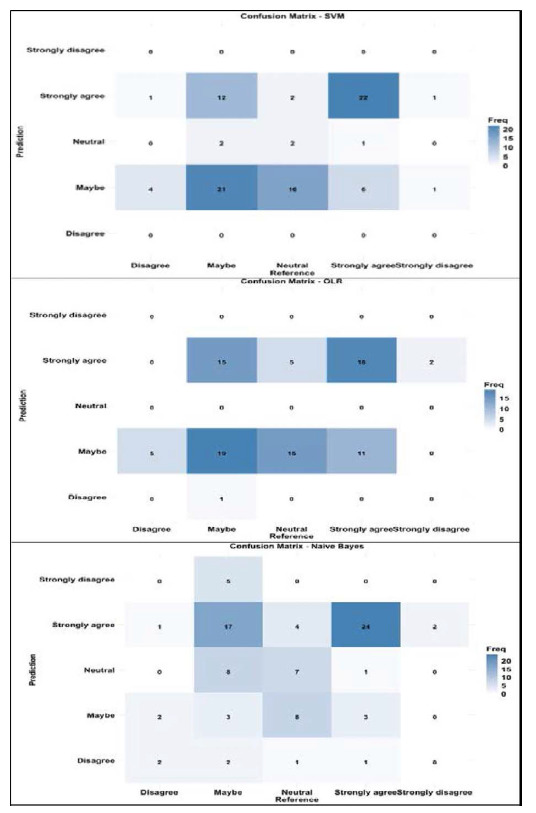
Confusion matrices of Likert scale levels for the different machine learning models.

Figure 1 presents the CM plots for the ML algorithms used, revealing a broader distribution of predictions across the categories, which indicates potential misclassification issues. The results of the ML analysis indicate that the OLR model is the best-performing algorithm for predicting the belief that HMCAS staff need to learn more languages.

**Figure 2.**
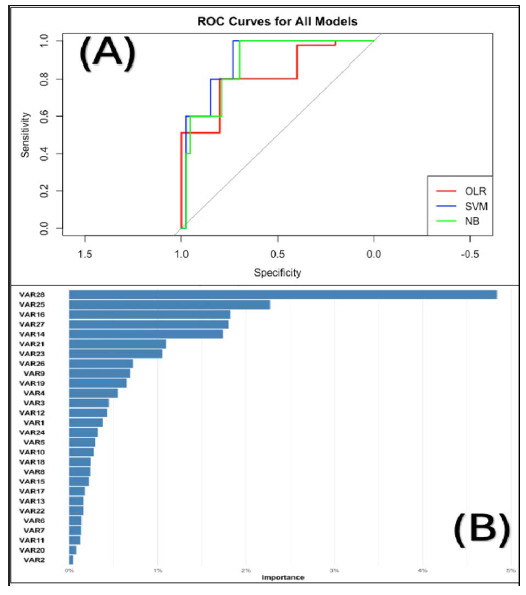
Receiver operating characteristic (ROC) curves for all machine learning models (A) and the feature importance plot for the support vector machine model (B).

Figure 2(A) shows the ROC curves for the different models. The curves of the different models exhibited a step-like progression. The more the curve plateaus at a higher value, the better the model identifies the “Strongly agree” cases across the items. Although the NB model showed a more extended plateau at a high value across many items, all models performed similarly in classifying the Likert scale categories. Therefore, the SVM was considered the best, achieving the highest AUC value of 0.84 for identifying the “Strongly agree” category.

Figure 2(B) and Table 4 (II) provide insights into the feature importance results of the SVM algorithm. The advantage of the ML feature importance technique was its ability to rank variables based on their predictive impact, thereby facilitating a clearer identification of the most influential factors compared to traditional SVM approaches. The standard SVM focuses on coefficient estimates and their statistical significance without providing a clear ranking of variable importance. In contrast, the ML feature importance technique provides a clear, quantitative ranking of predictors, identifying the most crucial ones and enhancing focused decision-making. In Table 4 (II), the letters “L” (Linear), “C” (Cubic), “Q” (Quadratic), and “^4” or “4th power” are used to represent the nonlinear effect of the continuous variables (Likert scores), enabling systematic modeling of the categorical variables based on their influential effect. In Figure 2(B) and Table 4 (II), the most influential factors in the SVM model were identified as the extreme ends of the Likert scale (VAR28), indicating that strong opinions on language barriers significantly impact the overall assessment. Moderate disagreement and a willingness to enhance language skills (VAR25 and VAR16) emerged as the next most crucial elements, emphasizing the importance of proactively addressing communication challenges. The frequency of encountering communication difficulties due to language barriers is regarded as moderate importance (VAR1, 3, and 4). In contrast, the variables associated with using family members as interpreters and the ability to attend language courses were considered to have relatively low importance (VAR6, 7, 11, and 20), suggesting that these practical considerations may have less influence on the overall evaluation of language barriers in the EMS setting.

## Discussion

Previous research has identified that communication barriers between emergency medical dispatchers and paramedics may lead to delays in patient access and the commencement of treatment.^21,22^ This study used advanced statistical and computational techniques to examine the opinions of frontline EMS personnel in Qatar and to identify potential strategies for improvement. ML techniques provided a data-driven method to identify and rank the most influential factors affecting staff perceptions of language-related challenges, thereby providing a more accurate representation of the ordered nature of staff views. This data-driven approach revealed patterns in staff opinions, such as predicting HMCAS staff perceptions about the need to learn more languages and its significant association with certain variables such as the frequency of experiencing communication challenges, the current multilingual capabilities of paramedics, and relying on bystanders for interpretation during medical emergencies. Healthcare organizations have the opportunity to develop evidence-based strategies to address language barriers more effectively, thereby improving patient care, reducing miscommunication and misdiagnosing risks, and enhancing the overall quality of pre-hospital medical services.^23^ Furthermore, the feature importance analysis results presented in Table 4 and Figure 2 identified the most significant aspects of language barriers from the perspective of EMS personnel. These findings advocate the implementation of targeted interventions such as language training programs or specialized communication tools. Recent studies have demonstrated that the interpreter apps can significantly improve satisfaction levels among both patients and medical staff.^24,25^ In summary, the application of ML analysis techniques to understand the opinions of staff and patients can assist healthcare organizations in developing actionable, evidence-based strategies to more effectively address language barriers, improve patient care, reduce miscommunication risks, and enhance the overall quality of EMS.

The impact of language barriers on pre-hospital emergency care has emerged as an increasingly significant concern across diverse EMS systems globally.^26–28^ Although this study primarily focuses on HMCAS, its findings are relevant to EMS systems operating in linguistically diverse communities worldwide. Language barriers in pre-hospital emergency environments can lead to misdiagnoses, posing significant risks to patient safety.^22^ The challenges and potential solutions identified in this study may serve as a framework for similar initiatives on a global scale. In EMS environments characterized by linguistic diversity, there is a pressing need to implement targeted language education programs for EMS personnel, focusing on high-frequency languages in the service area. Additionally, integrating technology and real-time interpretation services can provide immediate language support during emergencies.^29,30^ A recent article highlighted the promising role of artificial intelligence (AI)-based translation tools in medical settings, noting that AI translation models have shown excellent proficiency in handling general topics.^31^ This study further demonstrated that AI translation technologies could produce translations within a timeframe ranging from seconds to minutes. Another study identified the crucial role of AI translation technologies in managing linguistic challenges during the COVID-19 pandemic, noting that the use of remote interpreting surged to 39.69% during the initial lockdown periods, ultimately reaching 67.53% of total interpreting days by 2021.^32^

However, various studies have advocated a careful approach when implementing interventions to address language barriers in healthcare delivery. A recent systematic review revealed that while the implementation of interpreters in the medical field increased staff satisfaction, it also resulted in increased costs and prolonged treatment durations.^33^ Furthermore, the application of AI-based language translation apps in the MENA region faces significant challenges due to the linguistic complexity of the Arabic language. Most AI language models are trained on Modern Standard Arabic (MSA), which is primarily used in academic and official contexts, but rarely spoken in everyday communication.^34^ The vast diversity of Arabic dialects across the MENA region presents a major obstacle to accurate translation, as these dialects can significantly differ from MSA and each other in terms of vocabulary. Unlike the variations observed among English dialects, Arabic dialects can be mutually unintelligible, with terms and expressions varying wildly between regions.^6^ This linguistic diversity poses a significant challenge for non-Arabic-speaking healthcare providers who attempt to use AI-based translation tools, as these tools may fail to accurately capture specific terminology used by patients speaking in their local dialects. Future efforts should focus on developing AI language models that are specifically trained on individual Arabic dialects. This approach would require extensive data collection and analysis for each dialect, yet it has the potential to yield more accurate and contextually appropriate translations for healthcare providers in the MENA region. Therefore, recruiting and retaining staff with diverse linguistic skills is another solution that could enhance overall communication capabilities in EMS systems. This strategy is consistent with recent research that emphasizes the importance of a linguistically diverse healthcare workforce in improving patient outcomes and satisfaction.^35,36^ Furthermore, future research in HMCAS and similar Middle Eastern EMS should aim to collect data on the number of encounters in which patients and EMS providers do not share a common primary language, thereby quantifying the incidence of this communication challenge. Integrating these features into electronic patient care records in HMCAS will enhance the understanding of the complexities surrounding this issue and provide important insights for reviewing medico-legal cases, particularly regarding the influence of language differences on the accuracy of clinical information collected.

The application of ML methods in this study for analyzing language barrier survey data represents an advancement in survey analysis methodologies. While traditional statistical methods can yield valuable insights, ML presents several advantages. Firstly, ML algorithms such as the SVM can identify complex, nonlinear patterns in the data that may not be apparent in conventional analysis, such as hidden relationships, patterns, and influential factors on staff opinion, as evidenced in our data. Secondly, ML models can predict responses to specific questions based on other survey responses. This functionality allows for the identification of potential issues, the recognition of staff who may require additional support or training, and the implementation of proactive measures. Furthermore, the application of ML in the analysis of survey data is consistent with the emerging trend of data-driven decision-making in healthcare, thereby contributing to the new era of quality improvement and patient safety initiatives.

Furthermore, the principle of continuous improvement through regular assessment and refinement of language-related interventions is crucial to ensure their effectiveness over time. Continuous improvement is a key principle of Kaizen, which advocates staff engagement.^37^ The iterative nature of Kaizen aligns well with the dynamic challenges of linguistic diversity in emergency settings. Regular assessment and refinement of language interventions are essential to ensure that these solutions remain relevant and effective. This continuous cycle would allow EMS providers to adapt to changing demographics, linguistically diverse populations, and emerging communication needs.

## Limitations

The significant difference in sample sizes between APs (*n* = 309) and CCPs (*n* = 3) posed a challenge for statistical analysis. Although Fisher's exact test was used to address this imbalance, a more balanced sample size would have provided stronger comparisons between these groups, particularly given that, in our setting, CCPs predominantly communicate in English, unlike the APs. In addition, the cross-sectional design of the study restricts the ability to draw causal conclusions and limits longitudinal insights into language barrier challenges over time. Future research should aim for more balanced sample sizes between groups to address these limitations, potentially through stratified sampling techniques. A larger CCP sample would facilitate more meaningful comparisons and potentially reveal role-specific insights. Furthermore, longitudinal studies could monitor changes in language barrier perceptions and assess the long-term effectiveness of interventions. Implementing a mixed-methods approach, which includes qualitative interviews or focus groups, could also yield richer insights into the nuances of language barriers in EMS settings. Expanding the scope of the study to include multiple EMS providers from different regions would enhance generalizability of findings and enable cross-cultural comparisons in addressing language barriers in pre-hospital care.

## Conclusion

This study highlights the significant impact of language barriers on pre-hospital emergency care in Qatar, with implications for similar multicultural settings globally. The advanced ML techniques used in this study revealed significant associations between staff roles, communication difficulties, and the perceived necessity for language skills. By leveraging the willingness of HMCAS personnel to learn additional languages and using predictive models, EMS organizations can enhance the quality of care provided to patients. These findings lay the foundation for developing targeted interventions that can be widely implemented in EMS systems operating in linguistically diverse communities worldwide. Future research should evaluate the long-term impact of these strategies on patient satisfaction and outcomes, as well as explore innovative technologies to overcome language barriers in pre-hospital emergency care.

### Competing interests

The authors declare that they have no conflict of interest.

### Authors’ contribution

**HF:** Conceptualization, data analysis and validation, and manuscript preparation. **HK:** Data collection. **IH and MCK:** Manuscript review and data collection. **GA**, **JL**, and **LS:** Manuscript review and study oversight.

### Acknowledgments

We thank the HMCAS staff for their participation in the survey. We also thank the experts who contributed to the validation of the survey used in this study. Additionally, we thank the reviewers for their constructive feedback, which significantly enhanced the quality of the article.

### Ethical approval

This quality improvement study received approval from the HMCAS Group Quality, Patient Safety, and Risk Management department, under reference number QI-HMCASG-2023-006.

## Figures and Tables

**Table 1:** Descriptive analysis results of the demographic variables of the survey respondents.

I. Continuous variables		

Variable		*N* = 312^*^

	Mean (SD)	Median [IQR]

Age (years)	36.57 (5.38)	36 [34, 39]

Years working in the MENA region	8.90 (5.60)	8 [4, 11.31]

Years working in Qatar (patient-facing role)	7.20 (4.70)	7 [4, 9]

II. Categorical variables		

Variable	AP	CCP

Overall, *N*=312^*^	309 (99%)	3 (1.0%)

Egyptian, *N*=1^*^	1 (100%)	0 (0%)

Filipino, *N*=100^*^	100 (100%)	0 (0%)

Indian, *N*=73^*^	73 (100%)	0 (0%)

Jordanian, *N*=44^*^	44 (100%)	0 (0%)

Moroccan, *N*=1^*^	1 (100%)	0 (0%)

Other, *N*=1^*^	1 (100%)	0 (0%)

Palestinian, *N*=1^*^	1 (100%)	0 (0%)

South African, *N*=3^*^	0 (0%)	3 (100%)

Syrian, *N*=1^*^	1 (100%)	0 (0%)

Tunisian, *N*=87^*^	87 (100%)	0 (0%)


^*^Mean (SD); n (%).

**Table 2:** Content validity and reliability results of the survey items.

I. Cohen’s kappa results					

	Clarity of language	Relevant to the subject	Pertinence of the item	Good indicator	Average

Item 1	1	1	0	0	0.5

Item 2	1	0.61	0.61	-0.13	0.53

Item 3	0.51	1	0.78	0.16	0.63

Item 4	0.63	1	1	0.14	0.71

Item 5	0.36	0.75	1	-0.16	0.52

Item 6	1	0.63	1	0.63	0.82

Item 7	1	1	1	0.63	0.91

Item 8	0.63	1	1	0.08	0.66

Item 9	0.40	0.77	1	0.08	0.57

Item 10	0.33	1	0.75	-0.05	0.52

Item 11	0.81	1	1	0.63	0.87

Item 12	1	0.80	1	0.37	0.82

Item 13	0.84	1	0.69	0.07	0.66

Item 14	0.68	0.84	1	0.49	0.77

Item 15	0.36	1	0.73	0.60	0.68

Item 16	0.6	1	0.73	0.61	0.75

Item 17	1	1	1	NA	1

Overall average					0.69

II. Kendall’s W					

	Clarity of language	Relevant to the subject	Pertinence of the item	Good indicator	Average

Item 1	0.75	0.38	0.38	0.62	0.53

Item 2	0.84	0.50	0.42	0.53	0.57

Item 3	0.89	0.85	0.81	0.81	0.84

Item 4	0.85	0.59	0.49	0.57	0.62

Item 5	0.85	0.56	0.39	0.42	0.55

Item 6	0.77	0.77	0.77	0.80	0.78

Item 7	1.00	0.80	0.59	0.62	0.75

Item 8	0.88	0.61	0.57	0.68	0.69

Item 9	0.86	0.59	0.55	0.61	0.65

Item 10	0.69	0.66	0.55	0.66	0.64

Item 11	0.95	0.91	0.87	0.88	0.90

Item 12	0.92	0.77	0.69	0.77	0.79

Item 13	0.94	0.65	0.57	0.64	0.70

Item 14	0.96	0.83	0.67	0.64	0.78

Item 15	0.77	0.77	0.79	0.86	0.80

Item 16	0.79	0.78	0.73	0.84	0.79

Item 17	1	NA	NA	NA	1.00

Overall average					0.73

III. Cronbach’s alpha results					

Statistics		Cronbach’s a	Standardized a		Average inter-item correlation

Values		0.56	0.42		0.3


**Table 3:** Bivariate analysis of language barrier survey variables.

I. Bivariate analysis							

Variable	Fisher’s statistic	*p*	df	95% CI			

	Role within HMCAS (AP/CCP)						

Q6	9.74	0.05	4	(3.27, 29.63)			

Q4	6.46	0.17	4	(1.98, 24.22)			

Q5	2.89	0.6	4	(0.97, 17.72)			

Q2	4.47	0.35	4	(1.36, 20.72)			

Q1	3.8	0.43	4	(1.18, 19.48)			

Q3	3.57	0.47	4	(1.12, 19.03)			

Q7	2.14	0.71	4	(0.81, 16.19)			

II. Kruskal’s test analysis							

Variable	Kruskal-Wallis test results						

	Q1	Q2	Q3	Q4	Q5	Q6	Q7

Kruskal-Wallis H	0.66	0.73	0.01	5.93	0	2.94	0.6

*p* value	0.42	0.394	0.93	0.02	0.97	0.09	0.44

df	1	1	1	1	1	1	1

Significant pairs (Bonferroni)	NA	NA	NA	1	NA	NA	NA

Significant (*p* < 0.05)							

* No*	1	1	1	0	1	1	1

* Yes*	0	0	0	1	0	0	0


Q1: Are you willing to learn more languages to be more comfortable with providing care to a broader range of patients?

Q2: Do you believe that HMCAS staff with patient-facing roles need to learn more languages?

Q3: Given your current workload, do you believe that you are able to attend language courses provided by HMC?

Q4: How often have you experienced difficulties in communicating with patients due to language barrier?

Q5: In general, when facing a language barrier issue with a patient, how comfortable have you been with using a bystander family member as an interpreter?

Q6: In general, when facing a language barrier issue with a patient, how sufficient is the information you have managed to obtain from the patient to provide adequate care?

Q7: In relation to the FIFA World Cup Qatar 2022^TM^, how satisfied were you with your ability to communicate with fans?

**Table 4:** Machine learning analysis results of language barrier survey variables.

I. Metrics performance						

Model	Accuracy	Kappa	Sensitivity	Specificity	AIC	BIC

OLR	0.41	0.08	0.23	0.82	593.04	688.19

SVM	0.50	0.23	0.30	0.84	–	–

NB	0.40	0.16	0.333	0.83	–	–

	AUC (Maybe)	AUC (Neutral)	AUC (Strongly agree)	AUC (Disagree)	AUC (Strongly disagree)	

OLR	0.78	0.56	0.77	0.71	0.60	

SVM	0.79	0.68	0.84	0.77	0.66	

NB	0.55	0.72	0.82	0.88	0.77	

II. Feature importance variables identified for the SVM (plotted in Figure 2(B))						

VarLabels	Definition					

VAR1	How often have you experienced difficulties in communicating with patients due to language barrier?L					

VAR2	How often have you experienced difficulties communicating with patients due to language barriers?Q					

VAR3	How often have you experienced difficulties in communicating with patients due to language barrier?C					

VAR4	How often have you experienced difficulties communicating with patients due to language barriers?^4					

VAR5	In general, when facing a language barrier issue with a patient, how sufficient is the information you have managed to obtain from the patient to provide adequate care?L					

VAR6	In general, when facing a language barrier issue with a patient, how sufficient is the information you have managed to obtain from the patient to provide adequate care?Q					

VAR7	In general, when facing a language barrier issue with a patient, how sufficient is the information you have managed to obtain from the patient to provide adequate care?C					

VAR8	In general, when facing a language barrier issue with a patient, how sufficient is the information you have managed to obtain from the patient to provide adequate care?^4					

VAR9	In general, when facing a language barrier issue with a patient, how comfortable have you been with using a bystander family member as an interpreter?L					

VAR10	In general, when facing a language barrier issue with a patient, how comfortable have you been with using a bystander family member as an interpreter?Q					

VAR11	In general, when facing a language barrier issue with a patient, how comfortable have you been with using a bystander family member as an interpreter?C					

VAR12	In general, when facing a language barrier issue with a patient, how comfortable have you been with using a bystander family member as an interpreter?^4					

VAR13	Are you willing to learn more languages to be more comfortable with providing care to a broader range of patients?L					

VAR14	Are you willing to learn more languages to be more comfortable with providing care to a broader range of patients?Q					

VAR15	Are you willing to learn more languages to be more comfortable with providing care to a broader range of patients?C					

VAR16	Are you willing to learn more languages to be more comfortable with providing care to a broader range of patients?^4					

VAR17	Given your current workload, do you believe that you are able to attend language courses provided by HMC?L					

VAR18	Given your current workload, do you believe that you are able to attend language courses provided by HMC?Q					

VAR19	Given your current workload, do you believe that you are able to attend language courses provided by HMC?C					

VAR20	Given your current workload, do you believe that you are able to attend language courses provided by HMC?^4					

VAR21	In relation to the FIFA World Cup Qatar 2022^TM^, how satisfied were you with your ability to communicate with fans?L					

VAR22	In relation to the FIFA World Cup Qatar 2022^TM^, how satisfied were you with your ability to communicate with fans?Q					

VAR23	In relation to the FIFA World Cup Qatar 2022^TM^, how satisfied were you with your ability to communicate with fans?C					

VAR24	In relation to the FIFA World Cup Qatar 2022^TM^, how satisfied were you with your ability to communicate with fans?^4					

VAR25	Disagree|Maybe					

VAR26	Maybe|Neutral					

VAR27	Neutral|Strongly agree					

VAR28	Strongly agree|Strongly disagree					


**Table tbl5:** Table A. Count and percentages of responses to the language barrier survey by participants’ nationality.

		Nationality									

Variable	Overall, *N*=312^*^	Egyptian, *N*=1^*^	Filipino, *N*=100^*^	Indian, *N*=73^*^	Jordanian, *N*=44^*^	Moroccan, *N*=1^*^	Other, *N*=1^*^	Palestinian, *N*=1^*^	South African, *N*=3^*^	Syrian, *N*=1^*^	Tunisian, *N*=87^*^

Items	157 (90)	18 (NA)	142 (91)	172 (84)	167 (94)	148 (NA)	98 (NA)	169 (NA)	161 (100)	134 (NA)	157 (93)

What is your role within homes?											

AP	309 (99%)	1 (100%)	100 (100%)	73 (100%)	44 (100%)	1 (100%)	1 (100%)	1 (100%)	0 (0%)	1 (100%)	87 (100%)

CCP	3 (1.0%)	0 (0%)	0 (0%)	0 (0%)	0 (0%)	0 (0%)	0 (0%)	0 (0%)	3 (100%)	0 (0%)	0 (0%)

Age (years)	36.6 (5.4)	38.0 (NA)	35.4 (5.3)	34.1 (3.6)	37.9 (4.6)	47.0 (NA)	29.0 (NA)	56.0 (NA)	39.0 (9.5)	59.0 (NA)	38.7 (5.0)

Number of years working in the Middle East and North Africa region	8.9 (5.6)	15.0 (NA)	7.0 (4.8)	6.4 (3.4)	11.9 (5.7)	26.0 (NA)	3.0 (NA)	23.0 (NA)	4.3 (3.2)	25.0 (NA)	11.3 (5.6)

Number of years working in Qatar in an inpatient-facing role	7.2 (4.7)	10.0 (NA)	5.7 (4.5)	6.0 (3.4)	8.2 (4.2)	19.0 (NA)	3.0 (NA)	23.0 (NA)	4.0 (3.5)	25.0 (NA)	9.2 (4.4)

How often have you experienced difficulties in communicating with patients due to language barrier?											

Never	7 (2.2%)	0 (0%)	0 (0%)	3 (4.1%)	1 (2.3%)	0 (0%)	0 (0%)	1 (100%)	0 (0%)	0 (0%)	2 (2.3%)

Occasionally	127 (41%)	1 (100%)	51 (51%)	28 (38%)	10 (23%)	1 (100%)	0 (0%)	0 (0%)	0 (0%)	0 (0%)	36 (41%)

Rarely	97 (31%)	0 (0%)	16 (16%)	29 (40%)	22 (50%)	0 (0%)	0 (0%)	0 (0%)	0 (0%)	0 (0%)	30 (34%)

Undecided	7 (2.2%)	0 (0%)	1 (1.0%)	1 (1.4%)	2 (4.5%)	0 (0%)	0 (0%)	0 (0%)	0 (0%)	1 (100%)	2 (2.3%)

Very Frequently	74 (24%)	0 (0%)	32 (32%)	12 (16%)	9 (20%)	0 (0%)	1 (100%)	0 (0%)	3 (100%)	0 (0%)	17 (20%)

In general, when facing a language barrier issue with a patient, how sufficient is the information you have managed to obtain from the patient to provide adequate care?											

Insufficient	51 (16%)	0 (0%)	13 (13%)	6 (8.2%)	9 (20%)	0 (0%)	0 (0%)	0 (0%)	2 (67%)	0 (0%)	21 (24%)

Neutral	64 (21%)	0 (0%)	27 (27%)	16 (22%)	9 (20%)	0 (0%)	0 (0%)	1 (100%)	0 (0%)	1 (100%)	10 (11%)

Slightly sufficient	84 (27%)	1 (100%)	29 (29%)	13 (18%)	12 (27%)	1 (100%)	0 (0%)	0 (0%)	1 (33%)	0 (0%)	27 (31%)

Sufficient	80 (26%)	0 (0%)	30 (30%)	24 (33%)	6 (14%)	0 (0%)	0 (0%)	0 (0%)	0 (0%)	0 (0%)	20 (23%)

Very sufficient	33 (11%)	0 (0%)	1 (1.0%)	14 (19%)	8 (18%)	0 (0%)	1 (100%)	0 (0%)	0 (0%)	0 (0%)	9 (10%)

In general, when facing a language barrier issue with a patient, how comfortable have you been with using a bystander family member as an interpreter?											

Neither comfortable nor uncomfortable	36 (12%)	0 (0%)	12 (12%)	6 (8.2%)	3 (6.8%)	0 (0%)	1 (100%)	0 (0%)	0 (0%)	1 (100%)	13 (15%)

Not at all comfortable	19 (6.1%)	0 (0%)	5 (5.0%)	2 (2.7%)	8 (18%)	0 (0%)	0 (0%)	0 (0%)	0 (0%)	0 (0%)	4 (4.6%)

Somewhat comfortable	140 (45%)	0 (0%)	54 (54%)	27 (37%)	19 (43%)	1 (100%)	0 (0%)	0 (0%)	2 (67%)	0 (0%)	37 (43%)

Somewhat uncomfortable	36 (12%)	0 (0%)	11 (11%)	5 (6.8%)	3 (6.8%)	0 (0%)	0 (0%)	0 (0%)	1 (33%)	0 (0%)	16 (18%)

Very comfortable	81 (26%)	1 (100%)	18 (18%)	33 (45%)	11 (25%)	0 (0%)	0 (0%)	1 (100%)	0 (0%)	0 (0%)	17 (20%)

Do you believe that HMCAS staff with the patient-facing role need to learn more languages?											

Disagree	19 (6.1%)	0 (0%)	0 (0%)	3 (4.1%)	5 (11%)	1 (100%)	0 (0%)	0 (0%)	1 (33%)	0 (0%)	9 (10%)

Maybe	118 (38%)	0 (0%)	25 (25%)	23 (32%)	19 (43%)	0 (0%)	1 (100%)	0 (0%)	1 (33%)	1 (100%)	48 (55%)

Neutral	69 (22%)	0 (0%)	30 (30%)	17 (23%)	8 (18%)	0 (0%)	0 (0%)	1 (100%)	0 (0%)	0 (0%)	13 (15%)

Strongly agree	97 (31%)	1 (100%)	43 (43%)	28 (38%)	8 (18%)	0 (0%)	0 (0%)	0 (0%)	1 (33%)	0 (0%)	16 (18%)

Strongly disagree	9 (2.9%)	0 (0%)	2 (2.0%)	2 (2.7%)	4 (9.1%)	0 (0%)	0 (0%)	0 (0%)	0 (0%)	0 (0%)	1 (1.1%)

Are you willing to learn more languages to be more comfortable with providing care to a broader range of patients?											

Definitely	122 (39%)	0 (0%)	57 (57%)	46 (63%)	9 (20%)	0 (0%)	0 (0%)	0 (0%)	2 (67%)	1 (100%)	7 (8.0%)

Definitely not	10 (3.2%)	0 (0%)	1 (1.0%)	1 (1.4%)	0 (0%)	0 (0%)	0 (0%)	0 (0%)	0 (0%)	0 (0%)	8 (9.2%)

Neutral	78 (25%)	0 (0%)	25 (25%)	12 (16%)	12 (27%)	0 (0%)	1 (100%)	1 (100%)	0 (0%)	0 (0%)	27 (31%)

Probably not	31 (9.9%)	0 (0%)	2 (2.0%)	4 (5.5%)	9 (20%)	1 (100%)	0 (0%)	0 (0%)	1 (33%)	0 (0%)	14 (16%)

Very probably	71 (23%)	1 (100%)	15 (15%)	10 (14%)	14 (32%)	0 (0%)	0 (0%)	0 (0%)	0 (0%)	0 (0%)	31 (36%)

Given your current workload, do you believe that you are able to attend language courses provided by HMC?											

Agree	111 (36%)	1 (100%)	32 (32%)	24 (33%)	19 (43%)	0 (0%)	0 (0%)	1 (100%)	1 (33%)	1 (100%)	32 (37%)

Disagree	41 (13%)	0 (0%)	12 (12%)	8 (11%)	7 (16%)	0 (0%)	0 (0%)	0 (0%)	1 (33%)	0 (0%)	13 (15%)

Neutral	85 (27%)	0 (0%)	34 (34%)	21 (29%)	7 (16%)	1 (100%)	1 (100%)	0 (0%)	0 (0%)	0 (0%)	21 (24%)

Strongly agree	41 (13%)	0 (0%)	11 (11%)	12 (16%)	5 (11%)	0 (0%)	0 (0%)	0 (0%)	0 (0%)	0 (0%)	13 (15%)

Strongly disagree	34 (11%)	0 (0%)	11 (11%)	8 (11%)	6 (14%)	0 (0%)	0 (0%)	0 (0%)	1 (33%)	0 (0%)	8 (9.2%)

In relation to the FIFA World Cup Qatar 2022^TM^, how satisfied were you with your ability to communicate with fans?											

Dissatisfied	4 (1.3%)	0 (0%)	0 (0%)	0 (0%)	1 (2.3%)	0 (0%)	0 (0%)	0 (0%)	0 (0%)	1 (100%)	2 (2.3%)

Extremely satisfied	73 (23%)	0 (0%)	17 (17%)	31 (42%)	7 (16%)	1 (100%)	0 (0%)	0 (0%)	0 (0%)	0 (0%)	17 (20%)

Neither satisfied nor dissatisfied	23 (7.4%)	0 (0%)	13 (13%)	3 (4.1%)	4 (9.1%)	0 (0%)	0 (0%)	0 (0%)	0 (0%)	0 (0%)	3 (3.4%)

Satisfied	183 (59%)	1 (100%)	63 (63%)	31 (42%)	27 (61%)	0 (0%)	0 (0%)	1 (100%)	3 (100%)	0 (0%)	57 (66%)

Very dissatisfied	29 (9.3%)	0 (0%)	7 (7.0%)	8 (11%)	5 (11%)	0 (0%)	1 (100%)	0 (0%)	0 (0%)	0 (0%)	8 (9.2%)


^*^Mean (SD); n (%).

## Appendix

[Table tbl5]
